# Nature-Inspired Surface Engineering for Efficient Atmospheric Water Harvesting

**DOI:** 10.1021/acssuschemeng.3c00760

**Published:** 2023-07-18

**Authors:** Zihao Li, Luheng Tang, Hanbin Wang, Subhash C. Singh, Xiaoming Wei, Zhongmin Yang, Chunlei Guo

**Affiliations:** † The Institute of Optics, University of Rochester, Rochester, New York 14627, United States; ‡ School of Physics and Optoelectronics, South China University of Technology, Guangzhou 510640, China

**Keywords:** water harvesting, chemical-laser treatment, superhydrophobic/superhydrophilic surface, aluminum

## Abstract

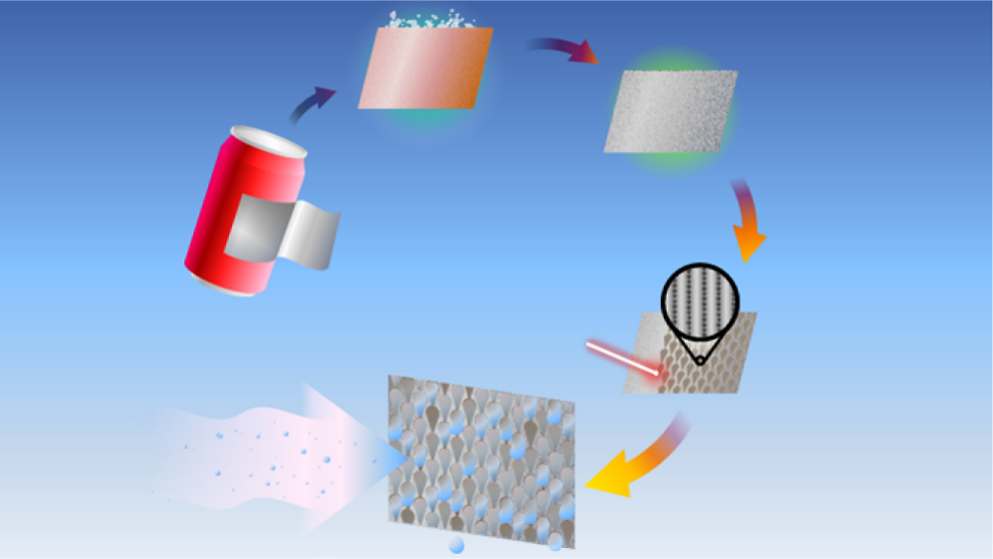

Atmospheric water harvesting is a sustainable solution to global water shortage, which requires high efficiency, high durability, low cost, and environmentally friendly water collectors. In this paper, we report a novel water collector design based on a nature-inspired hybrid superhydrophilic/superhydrophobic aluminum surface. The surface is fabricated by combining laser and chemical treatments. We achieve a 163° contrast in contact angles between the superhydrophilic pattern and the superhydrophobic background. Such a unique superhydrophilic/superhydrophobic combination presents a self-pumped mechanism, providing the hybrid collector with highly efficient water harvesting performance. Based on simulations and experimental measurements, the water harvesting rate of the repeating units of the pattern was optimized, and the corresponding hybrid collector achieves a water harvesting rate of 0.85 kg m^–2^ h^–1^. Additionally, our hybrid collector also exhibits good stability, flexibility, as well as thermal conductivity and hence shows great potential for practical application.

## Abstract 2

Aluminum foil can be recycled to fabricate an efficient superhydrophilic/superhydrophobic hybrid water collector through combining laser and chemical treatments.

## 1 Introduction

The global water crisis is continuously increasing due to the combined effect of climate change, population increase, deforestation, and rapid industrialization and urbanization. Over 2 billion people are currently living in water-stressed regions, and the number is projected to increase to over 5 billion by 2050.^1^ The atmosphere is a significant source of water, which can store up to 4% of water vapor at 30 °C. If harvested efficiently, atmospheric water harvesting (AWH) can provide a decentralized solution to the ever-growing water crisis. Water can be generated at the locations of its end-users, and thus AWH can solve the issue of long-distance transportation to deliver water in rural areas. In regions with low precipitation, AWH can be used to harvest water for the daily life of the residents as well as for irrigation.^2^ Additionally, AWH can reduce the content of water vapor, a greenhouse medium, in the atmosphere to reverse global warming.^3^

Owing to the importance of AWH, extensive research efforts have been devoted to developing water collectors based on different working principles. The early collectors were relatively simple—usually meshes with a large surface area without any microstructures, but they exhibited low water harvesting efficiency.^4^ In nature, countless plants and animals inhabiting arid climates have evolved to accommodate the harsh environment. Either their skin or the secretion they discharge possesses special microstructures that help the surface to maximize the capillary effect, heterogeneous wettability, or Laplace pressure gradient to efficiently harvest water from the atmosphere.^5^ Following the studies of biotic water condensation and transportation, many artificial bio-inspired materials for AWH have been developed,^6^ including spindle-knots that imitate the spider silk of *Uloborus walckenaerius*,^7^ non-parallel plates motivated by the feeding mechanism of *Phalaropus*,^8^ conical wires with gradient wettability^9^ and coated nanoneedles^10^ enlightened by the spines of *Opuntia microdasys*, surface with inclined arc pitted microchannels that imitate the peristome of *Nepenthes alata*,^11^ three-dimensional hierarchical structure that shares the same feature with the leaves of *Cotula fallax*(12) or hair of *Salsola crassa*,^13^ and hybrid hydrophobic/hydrophilic patterned surface that mimics the back of *Stenocara gracilipes*.^14^ Among these materials, the hybrid surface that possesses self-pumped droplet-delivering ability is suggested to be one of the most efficient designs.

The most important characteristic of a collector for AWH is that it can condense water vapor from the atmosphere with high efficiency and release the condensed water droplets from its surface in a frequent manner. Biomimetic hybrid collectors composed of a hydrophobic background and a hydrophilic pattern show promising performance in finishing both tasks. The core of the hybrid collector is to continuously gather water to its hydrophilic region so that water droplets can quickly reach their critical mass (the maximum mass of the droplet that can be held on the hydrophilic area of the surface) to sustain a high renewal rate of the surface. This function should be present in both gas and liquid phases. In the gas phase, water vapor will be repelled by the hydrophobic region of the hybrid collector, leading to a rise in the local vapor concentration around the hydrophilic region. The higher vapor concentration over the hydrophilic region promotes filmwise condensation. On the other hand, in the liquid phase, the water droplets formed in the hydrophobic region through dropwise condensation experience lower surface drag, and therefore, these droplets can be easily transported from the hydrophobic region to the hydrophilic region under the effect of gravity, airflow, or coalescence.^15^ As a result, rather than spreading through the whole surface of the collector, water will be mostly converged to and constrained within the hydrophilic region, which limits the area of the interface between water and air and reduces the re-evaporation from the surface.

Unfortunately, the currently available techniques of constructing hydrophilic/hydrophobic hybrid structure collectors present several disadvantages—these designs are either not long-lasting enough or too complicated for large-scale fabrication. For instance, several proposed collectors were made of polymeric materials,^16^ but these materials are poor thermal conductors and known for aging, degrading, as well as creeping,^17^ especially in high-temperature environments and under exposure to UV light.^18^ Some collectors combined two or more different materials to obtain the hybrid architecture;^19^ however the weak connection between the two materials deteriorates the structural stability of the collector. During the fabrication process of some other collectors, a mask was required to selectively modify the uncovered material and endow it with different properties.^20^ Consequently, despite the huge existing value and potential in application, few AWH collectors are commercially accessible,^21^ leaving a non-negligible gap between the need and supply.

**Figure 1:**
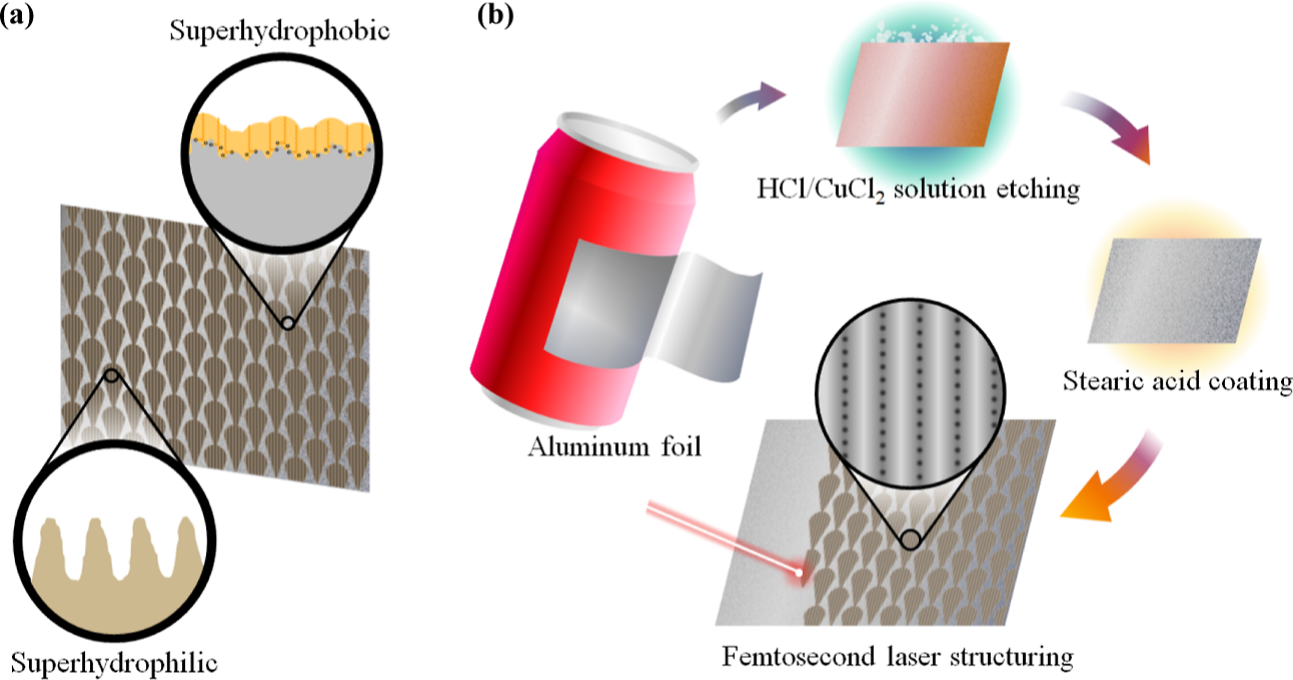
(a) Schematic diagram of the hybrid collector, including zoom-in cross-sectional views of the superhydrophobic background (top) and the superhydrophilic pattern (bottom). (b) Schematic illustration of the fabrication sequence: (1) Al foil being cut into pieces as raw material. (2) Etching of the Al foil to obtain a surface with microroughness. (3) Stearic acid-coating of the etched Al to form a superhydrophobic background. (4) Selective fs-laser-ablation to create a superhydrophilic pattern (zoom-in is the top view of the microchannels and self-organized microhole array introduced by fs-laser).

Recently, laser has been applied to fabricate composite AWH collectors by selectively removing one of the materials to generate a hybrid surface,^22^ drilling holes to connect the inside and outside of a bucket^23^ and scanning metal foams to form Janus membranes;^24^ laser deposition has also been used to transfer patterns from one material to another to create hybrid surfaces.^25^ To address the as-mentioned issues, selective laser surface ablation of aluminum (Al),^26^ copper (Cu),^27^ and poly(methyl methacrylate) (PMMA)^28^ has been applied to fabricate hybrid collectors. Although these designs tried to enhance the centralization of water and maximize the surface energy and Laplace pressure gradients, none of them focuses on minimizing the interfacial force between water droplets and the collector. Herein, we report a novel water collector for AWH based on a hybrid hydrophobic/hydrophilic Al surface, which aimed at interfacial force reduction and unidirectional transportation by optimizing the pattern of the shape of the superhydrophilic unit, as illustrated in Figure 1a. Al foil is chosen as a start because it is easily accessible, machinable, resists to aging, and has good thermal conductivity. The preparation procedures are presented in Figure 1b: the surface of Al is etched chemically and modified with stearic acid (Figure S1a) to form a superhydrophobic background and then selectively patterned with microstructure using a femtosecond (fs)-laser (Figure S1b). It has been reported that highly stable superhydrophobic functionality can be achieved through a simple and low-cost method of coating the surface with nonpolar group-terminated surfactant molecules,^29^ such as forming a stearic acid self-assembled monolayer.^30^ The stearic acid monolayer significantly reduces the water affinity of the Al surface. Its molecules connect to the Al substrate through covalent bonds, which are much stronger than van der Waals force, and therefore the collector is more robust and durable, poses no contamination threat, and can withstand a harsh environment.^31^ Fs-laser has an advantage in introducing stable micro/nanostructures onto different materials.^32^ A program-driven fs-laser scanner with a predesigned digital pattern is employed to create superhydrophilic patterns on the superhydrophobic background so that the whole scanning process can be done automatically, and no mask is needed. A comparison of our work and the related designs is shown in Table S1.

The developed hybrid AWH collector with superhydrophilic patterns surrounded by the superhydrophobic background (hybrid collector; hereafter) is bio-inspired by the surface of the Stenocara beetle’s elytra, which comprises a waxy background and randomly distributed smooth hydrophilic bumps with irregular shapes.^33^ However, nature still leaves plenty of room for us to further exploit its design to enhance the AWH performance. It is desirable to improve the design of the hybrid surface to assure that an even higher surface renewal rate can be achieved. Here, the parameters for laser scanning and the shapes of the hydrophilic pattern are optimized by combining both simulation and experimental methods to enhance its superwicking and superhydrophilic properties as well as reduce the critical mass of water that can be constrained within each repeating superhydrophilic unit of the pattern. Water harvesting rates were also measured to demonstrate the more frequently regenerated surface and the improved AWH performance of our hybrid collector with the optimized pattern.

## 2 Results and Discussion

### 2.1 Design and Optimization of the Superhydrophilic Pattern

The fast nucleation-coalescence-removal cycle reflects the superiority of the hybrid surface to the others, and thus, developing a collector with hybrid superhydrophilic and superhydrophobic characteristics is necessary. To reduce the difficulty in scaling up the collector, the hybrid collector is designed to be a combination of a continuous superhydrophobic background and an array of identical superhydrophilic units with the same size and shape. We combined both simulation and experimental methods to study the effect of the size and shape of each repeating unit, starting from a circle, on the efficiency of AWH. To maximize the efficiency of the hybrid collector, we started by focusing on only one unit of the repeating pattern to simplify the problem.

The degree of retention that a droplet on the collector receives can be described by the retention factor *r*(34)
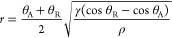
1where θ_A_ and θ_R_ are the critical advancing and receding contact angles (as illustrated in Figure S2a), respectively, while γ and ρ are the surface tension and density of water, respectively. Equation 1 shows that the droplet needs to have a larger difference between θ_A_ and θ_R_ to balance the retention and to start sliding on a hydrophilic surface. Based on this deduction, it is straightforward that a circle, being the only two-dimensional shape that owns infinite symmetry, corresponds to a droplet with an identical contact angle along the contour of the superhydrophilic unit. We can also conclude that, by introducing asymmetry to the circular unit, a larger disparity between θ_A_ and θ_R_ can be obtained, and the retention of the droplet will therefore decrease. The infinite symmetry of a circle can be broken by simply introducing an apex to its contour. This will reduce the axis of symmetry of the shape to 1. We set the two sides of the apex as tangent to the circle at the two intersections to ensure a smooth transition between the arc and the apex. Hereafter, this shape will be called a teardrop, and the schematic illustration of which is shown in Figure S2b.

**Figure 2:**
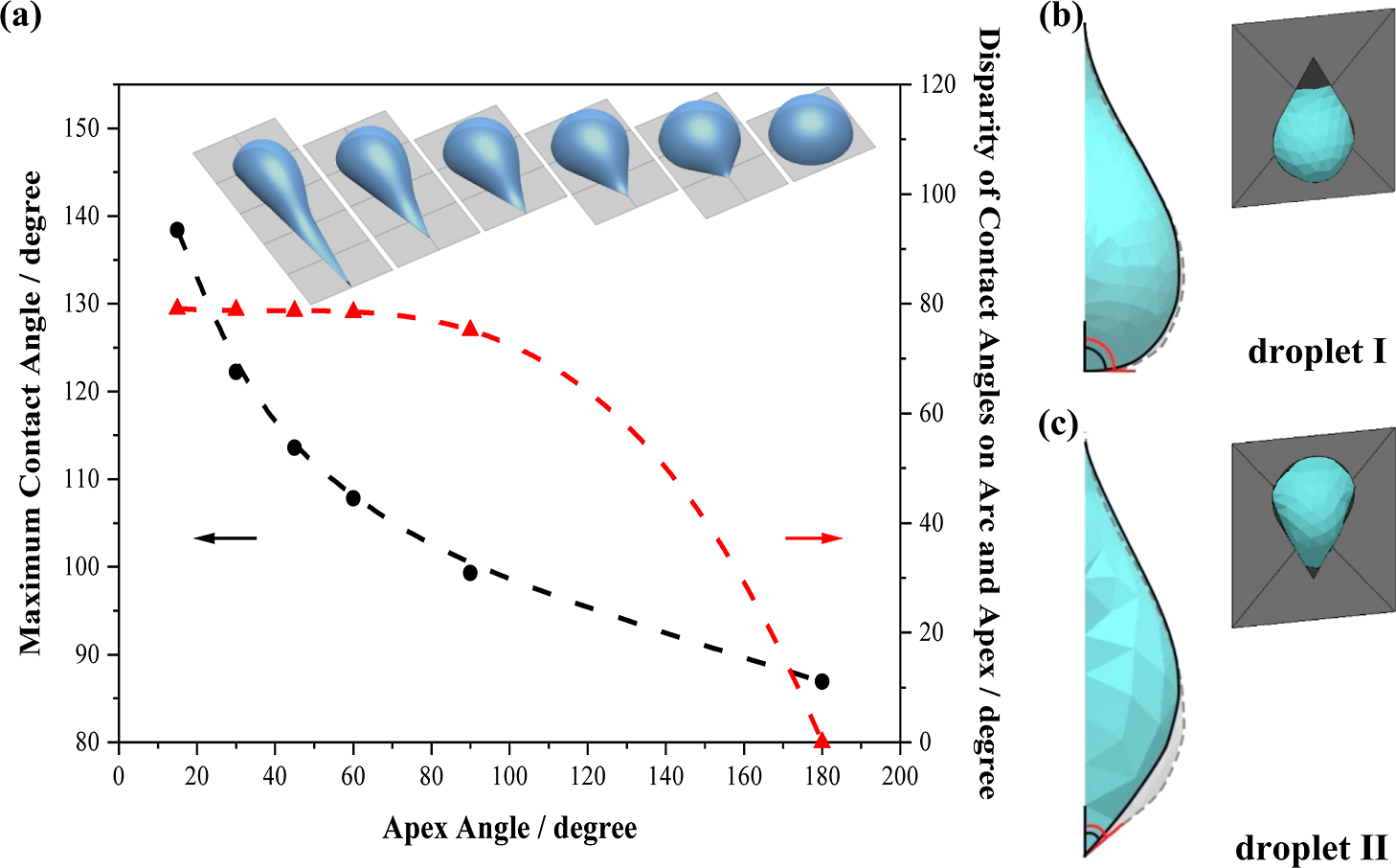
Simulations of the shape of water droplets. (a) Superhydrophilic units with the same area of 10 mm^2^ but different apex angles of 15, 30, 45, 60, 90, and 180° and the maximum contact angle of a water droplet with the mass of 20 mg that is constrained inside the corresponding unit. Apex angle versus maximum contact angle: disparity of contact angles on the arc and apex side of the unit of each droplet are shown in black and red with fitted curves (dashed lines). Side view of water droplets with mass of 50 mg constrained inside units with the shape of (b) erected teardrop and (c) inverted teardrop with fixed apex angle of 60° and area of 50 mm^2^ on a vertically placed collector with and without vertical disturbance (shown in blue and gray, respectively).

We first studied the influence of the additional apex on the superhydrophilic unit with the area of the unit set as constant. This part of the simulation was based on the assumption that the amount of water condensed on the surface is proportional to the area of the superhydrophilic units, while the superhydrophobic background has no contribution to water harvesting. Thus, the volume of the droplet is constant throughout the simulation. The simulation program HyDro100 was applied to study the contact angle of static water droplets placed on the superhydrophilic unit. By performing an energy-minimization calculation, the stable state of a water droplet that is constrained inside the pattern can be simulated. All HyDro100 simulations were done with the collector placed horizontally, which means gravity is perpendicular to its hybrid surface, and thus no horizontal external force will affect the shape of the droplet on the collector. After adding an apex to the circle, the contact angle of the droplet will no longer be equal around the contour of the unit. Our simulation reveals that the contact angle has a global minimum at the apex of the contour, and it reaches its global maxima at both shoulders of the apex (Figure S3). As shown in Figure 2a, when the area of the unit is kept constant, the contact angle maxima increase with the decrease in the apex angle. Decreasing the apex angle results in an increase in the disparity between the contact angles at the arc side and at the apex. Consequently, the teardrop shape is selected to be the initial shape of the repeating unit.

After designing the shape of the hydrophilic pattern, gravity is taken into consideration. In this step, all investigations will be based on the collector being placed vertically, which is the same way that the collector is placed in working conditions, and therefore the influence of gravity on the motion of the droplet cannot be ignored. In an ideal model represented by eq 2,^35^ for a static droplet on a vertical surface, the gravitational force (*F*_g_) on the droplet numerically equals the interfacial force between the droplet and the surface (right side of the equation) while they are opposite in direction
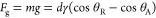
2where *m* is the mass of the droplet, *g* is the gravitational acceleration, and *d* is the maximum diameter of the contact area between the droplet and the repeating unit. The simulation program Surface Evolver was used to study how gravity shapes the surface of two droplets (denoted droplet I and droplet II) that are constrained within two units on the surface of two standing collectors. The area and apex angle of the hydrophilic units and the volume of the droplet are set to be 50 mm^2^, 60°, and 50 mg, respectively. The efficiency of the collectors was simulated in two opposite configurations, i.e., with the apex of the unit pointing upward (droplet I) and downward (droplet II) (as shown in the top-right insets of Figure 2b,c, respectively) to judge which direction is better at promoting the movement of the droplet. The parameter of gravitational acceleration was increased from 9.81 m s^–2^ by 10% in the simulation to mimic a vertical disturbance on the droplet. As shown in Figure 2b,c, the term (cos θ_R_ – cos θ_A_) of droplet I and II is changed by 11.7 and 61.5% after the disturbance was introduced, respectively. Hence, the disturbance imposes a stronger impact on droplet II and more interfacial force is needed to balance out the disturbance, otherwise, the droplet will begin to slide. In addition, for a given area and apex angle, our experiment also revealed that the critical mass of the water droplet constrained within the erected teardrop-shaped unit (droplet I) is much less than that of the droplet constrained inside an inverted teardrop-shaped (droplet II) unit. This result shows that the inverted teardrop has a lower retention than the erected one, which is beneficial to the regeneration of the surface.

During the accumulation of water within the inverted teardrop-shaped unit, the center of gravity of the constrained droplet slowly moves toward the apex of the unit. The two sides of the apex work as two blades of a scissor and exert shear force on both sides of the droplet to remove it from the surface. When the condensed water will slide down from the unit, its contact area with the superhydrophilic unit will decrease; however, the contact area with the hydrophobic background will increase, which in turn will gradually reduce the interfacial force between the collector surface and the water droplet. Since the arc is at the upper part of the unit while the apex angle is at the lower part, the difference in contact angle will be even greater when the collector is placed vertically in its working state, and the larger the maximum contact angle is, the condensed water is more likely to roll off from the surface.

The simulation results indicate that the superhydrophilic pattern with inverted teardrop-shaped units is the most suitable candidate for further optimization. Since area and apex angle are the only two parameters that are needed to define a teardrop shape, we aim to find the most compatible pair of these parameters through experimentation.

### 2.2 Fabrication and Characterizations

**Figure 3:**
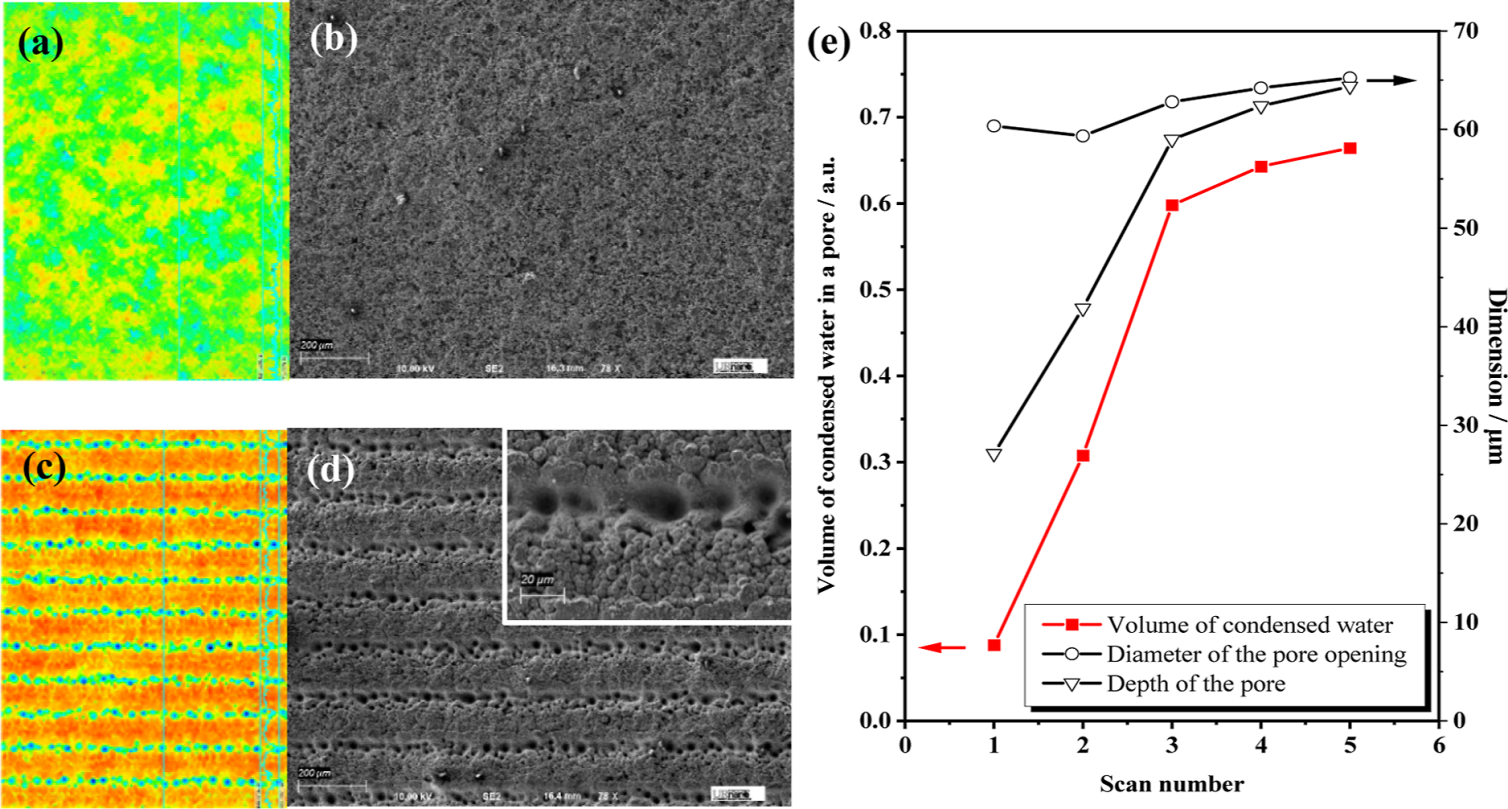
(a) 3D CLSM map and corresponding (b) SEM image of the superhydrophobic region of the hybrid collector. (c) CLSM map and corresponding (d) SEM image of the superhydrophilic region of the hybrid collector. Different colors in the elevation maps from blue to red only represent the relative elevation from low to high. (e) Effects of the fs-laser scan numbers on the diameters and depths of the microholes along with the volume of the condensed water in a microhole at equilibrium state.

The surface microstructure of different Al samples was characterized by confocal laser surface microscopy (CLSM) and scanning electron microscopy (SEM), and the results are shown in Figure 3. The untreated Al foil possesses a smooth surface. After chemical etching, the surface of Al becomes rough (Figure 3a) and uniform sponge-like villous nanostructures can be seen (Figure 3b), as a result of the intense attack of acid during etching. Despite having the same apparent surface area, the etched Al presents a much larger effective surface area owing to the nanostructures, which paves the way for tuning of the surface energy of Al toward the extreme. Irradiated by the fs-laser, parallel straight microchannels are ablated on the Al surface (Figure 3c). The morphology of the surface around the microchannels is constructed by microstructures covered with nanoprotrusions and nanocavities formed by melting and refreezing of the metal. At the bottom of each microchannel, there is a string of self-organized microholes introduced by laser ablation-induced incubation (Figure 3d).^36^ However, we reproduced the microhole array with only 2–3 scans (Figure S4), much less than the minimum required scan number reported, which might be due to the higher laser fluence used.

Formation of the self-organized microhole array is believed to take place only under fs-laser irradiation due to the localized melting within the small heat-affected zone of fs-laser, accompanied by the Marangoni effect. These microholes are important in enabling capillary condensation where vapor-phase water molecules get confined, resulting in an enhanced van der Waals interaction among them. The enhanced interaction causes condensation of water vapor below the saturation vapor pressure. As soon as the water gets condensed into the microholes, it forms an extended meniscus at the air–water interface that sets an equilibrium below the saturation vapor pressure, as suggested by the Kelvin equation (eq S5).^37^ Since this phenomenon takes place easier in smaller pores, the tenability of the microholes can be exploited to achieve better condensing property of the fs-laser-ablated Al. As shown in Figure S4, with accumulating scan numbers, the depth of the microholes increases and their tips become sharper, which is a favorable condition for capillary condensation. Since the self-organized microholes share a similar conical structure and the diameter of their opening is almost independent of the scan number, therefore for given experimental conditions, microholes with a sharper apex angle will condense more water (Figure 3e; eq S6). The depth and diameter of the pores increase with the scan number and stagnate at 5 scans, and thus this scan number is chosen for fs-laser fabricating of the superhydrophilic units.

Chemical etching is used to create microroughness on the surface of the Al foil to increase the surface area in the fabrication of the superhydrophobic background. After etching, not only the surface morphology but also the surface chemistry has been changed. As shown in Figure S5, the formation of hydroxyl groups on the surface of Al during chemical etching is suggested by the broad stretching band (νOH) centered at 3420 cm^–1^, which increases the water affinity of the surface and provides active sites for the carboxyl group of stearic acid to bond with.^38^ After treating the etched Al with stearic acid, a self-assembled stearic acid monolayer was engrafted on the surface (as illustrated in Figure 1a). Several characteristic peaks emerged after the stearic acid modification, including peaks at 1459, 2852, and 2925 cm^–1^, which are assigned to scissoring, symmetric, and asymmetric stretching of the methylene group in the alkyl chain of stearic acid (δ_s_CH_2_, ν_s_CH_2_, and ν_as_CH_2_), respectively.^39^ The peak at 1577 cm^–1^ is assigned to asymmetric stretching of the carboxylate group (ν_as_COO^–^) while no sign of the carboxyl group can be found, which is indicative of no free stearic acid being left on the surface.^40^ The formed insoluble aluminum stearate firmly anchors the stearic acid onto the Al surface through a covalent ester bond,^41^ and the nonpolar long alkyl chains are forced to face outward (Figure S5a) and thus the surface energy of the sample is reduced. Because of the ultralow surface energy of the superhydrophobic Al, water on the surface tends to contract, forming a Cassie–Baxter state droplet and leaving a minuscule interfacial area beneath it. The air trapped between the micro-protrusions weakens the interfacial force between the water droplet and the surface. These contributors provide the Al surface with excellent superhydrophobicity.

Fs-laser treatment can selectively turn the surface superhydrophilic by modifying the selected area with the formation of microgrooves/microholes and the simultaneous removal of the steric acid monolayer. It is evidenced by identical FTIR spectra of the etched and the laser-ablated Al (Figure S5b). The band centered at 880 cm^–1^ is assigned to the stretching of the aluminum–oxygen bond (νAl–O) of Al_2_O_3_.^36^

**Figure 4:**
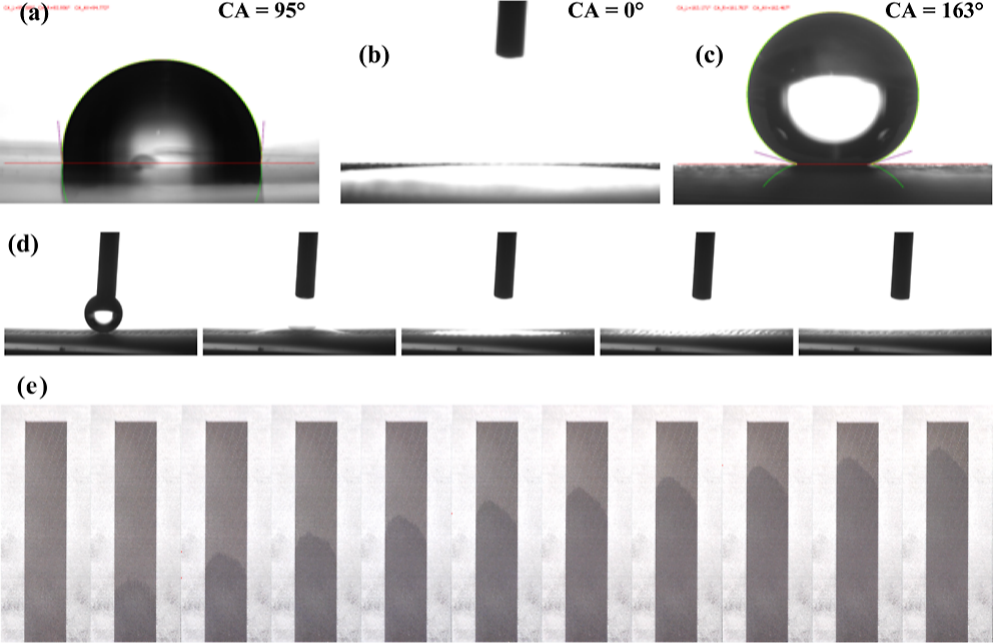
Contact angles of a water droplet on (a) untreated, (b) chemically-etched, and (c) stearic acid-coated Al samples. (d) Frames taken from a high-speed camera video of a water droplet at 0, 5, 10, 50, and 100 ms (from left to right) after it touched the surface of a horizontally placed fs-laser-treated Al sample. (e) Time-series snapshots of positions of the waterfront on a vertically mounted fs-laser-ablated Al foil with its microchannels parallel to the direction of gravitational force and its lower end brought in contact with the water surface. The time interval between two neighboring frames is 0.25 s (from left to right). The line spacing and the scan number are 0.15 and 5, respectively. The red dashed lines are indication of the waterfront.

To evaluate the wettability of the untreated, chemically etched, stearic acid mono-layered, and fs-laser-treated Al samples, the contact angles of a water droplet on these surfaces were measured. It can be found that the water on the untreated Al surface displays a contact angle of 95° (Figure 4a), representing that its surface is slightly hydrophobic. Although coating the material’s surface with nonpolar group-terminated molecules is an effective way to lower the surface energy, it is known that, even after reducing the surface energy to the theoretical minimum by coating only, the maximum contact angle of a water droplet on a smooth flat surface that can be reached is merely ∼120°.^42^ Therefore, chemical etching was applied to enlarge the surface area, since changing surface area is the only way to modify the wettability when the surface chemistry stays unchanged, as the Cassie–Baxter equation predicts.^43^ After etching Al, the contact angle shrinks to 0° (Figure 4b), indicating that the enlarged surface area drastically raises the surface energy and thus will be able to compensate for the consumption of surface energy during the spreading of water over the surface. After being coated with stearic acid, the water-repelling property of the etched Al is also enhanced consequentially, and a large contact angle of 163° can be obtained (Figure 4c).

Recently, we have reported the development of a superhydrophilic Al surface treated using almost unanimous fs-laser parameters that exhibits a superwicking property and water evaporation performance higher than an ideal evaporator working at 100% efficiency.^44^ Fs-laser treatment induces self-organized microholes, further enlarging the specific surface area to enhance the wettability of Al. Water spreads even faster within the rehydrophilized fs-laser-ablated area over the chemically etched and coated Al, which can be ascribed to the directional superwicking of the microchannels created by the fs-laser. This special wetting property of the fs-laser-ablated Al was demonstrated by putting a droplet of water onto the sample. As shown in Figure 4d, it takes less than only 0.1 s for the droplet to infiltrate into the microchannels and spread throughout the surface of a horizontally placed fs-laser-treated Al sample. The strong wettability is attributed to the parallel array of microchannels ablated on the surface, enabling a strong directional capillary effect.^45^ This is supported by Figure 4e, an observation of the anisotropic pervasion behavior of a water droplet’s waterfront, which shows the fast spreading speed of the waterfront along the direction of the microchannels. Superwicking and directional transportation are important properties that can be utilized by the hybrid collector so that the removal of the condensed water on the collector can be promoted. The significant contrast in wettability between the superhydrophilic and superhydrophobic regions established on the Al surface by combining chemical and fs-laser treatments is the foundation of an efficient water collector and the hybrid superhydrophilic/superhydrophobic collector can be further improved by combining the delicate optimization of the superhydrophilic pattern.

To ensure that the best wicking property of the superhydrophilic pattern can be achieved, the parameters (period and depth) of the parallel straight microchannels were optimized by studying the water spreading speed on the surface of vertically mounted superhydrophilic Al samples. The superhydrophilic strips consisting of parallel microchannels with line spacings of 0.1, 0.125, 0.15, 0.175, and 0.2 mm were fabricated on superhydrophobic Al foils. As presented in Figure S6a, when the lower ends of the samples touched the water surface, the water quickly climbed up through the capillary effect of the microchannels on every superhydrophilic strip. It is clear that the waterfronts elevate at different speeds and a faster elevation speed indicates a stronger wicking property of the strip. The sample with a microchannel line spacing of 0.15 mm showed the highest speed of the elevating waterfront. Furthermore, microchannels with different depths were created by varying the scan numbers of 1, 2, 3, 4, and 5 for laser ablation. As shown in Figure S6b, the wicking property of the superhydrophilic strips improves with increasing microchannel depths while the increment gets smaller after the scan number surpasses 3, which aligns with the prediction made earlier by measuring the cross-sectional profile of the samples. Therefore, the line spacing of 0.15 mm and the scan number of 5 were chosen for the fabrication of the designed and optimized inverted teardrop-shaped superhydrophilic patterns on the superhydrophobic background sample in sample fabrication in the following experiments.

### 2.3 Nucleation, Growth, and Release of the Water Droplets

To demonstrate the water harvesting superiority of the hybrid superhydrophilic/superhydrophobic collector over the other samples (untreated Al, completely superhydrophobic Al, and completely superhydrophilic Al), the nucleation, growth, and release dynamics of water droplets were recorded through a microscope with a setup shown in Figure S7.

**Figure 5:**
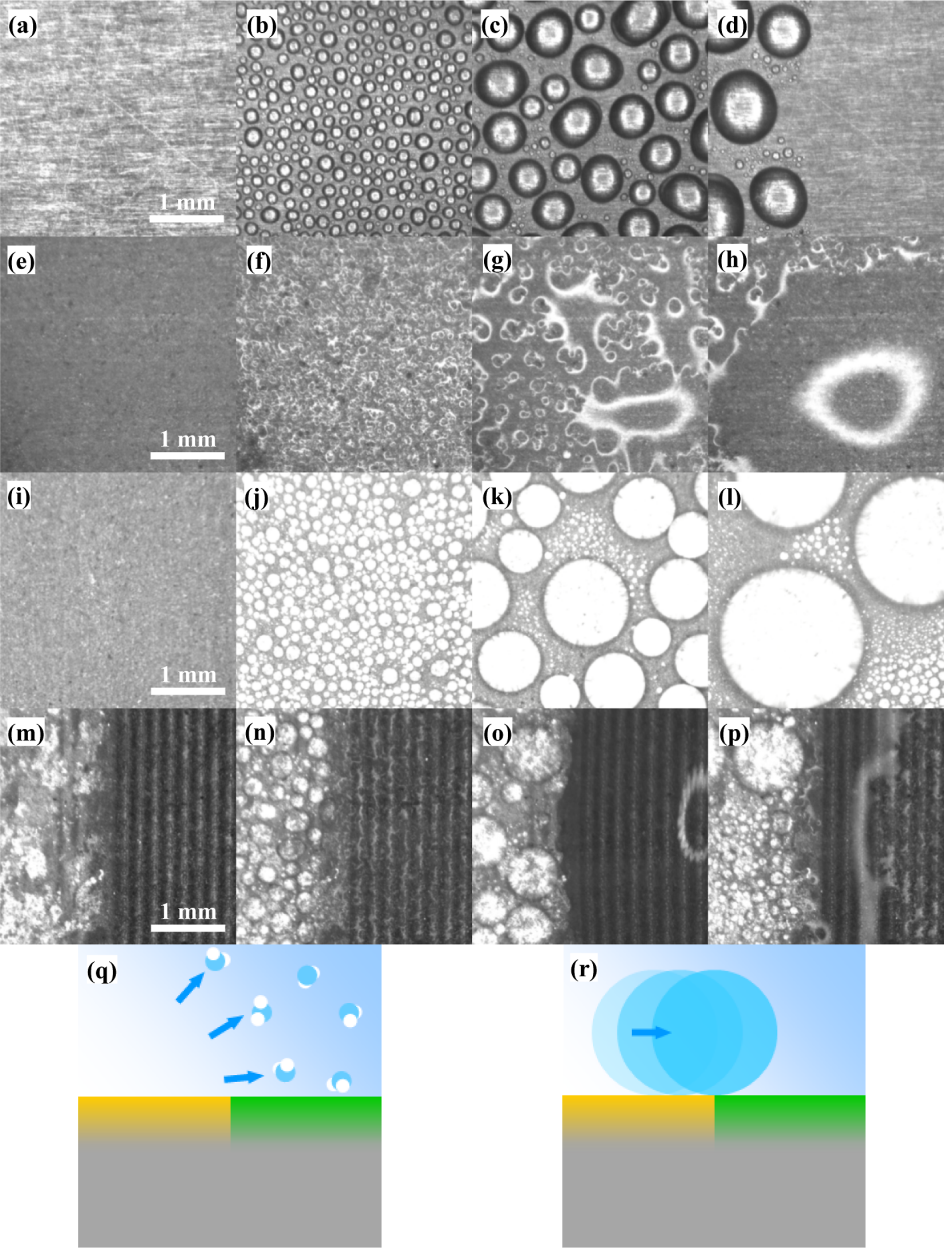
Microphotos recorded during the water condensation and regeneration process on (a–d) untreated, (e–h) completely superhydrophilic, (i–l) completely superhydrophobic, and (m–p) hybrid superhydrophilic/superhydrophobic Al. Schematic illustration of the two major water harvesting mechanisms of the hydrophobic/hydrophilic hybrid water collector take place in (q) gas phase and (r) liquid phase shown in cross section. Hydrophobic and hydrophilic regions of the collector are shown in yellow and green, respectively.

The images in the first column from the left (Figure 5a,e,i,m) are initial photographs of the clean samples before blowing vapor on them. The second, third, and fourth columns (from left to right) are the typical phases representing the nucleation, coalescence, and removal processes of water droplets on each sample. At the beginning of condensation, water nucleates and forms discrete droplets on the untreated Al (Figure 5b) and the superhydrophobic Al (Figure 5j) samples. However, it forms a thin film on the superhydrophilic Al surface (Figure 5f). The droplets on the untreated (Figure 5c) and superhydrophobic Al (Figure 5k) surfaces grow larger, while the water layer on the superhydrophilic Al (Figure 5g) becomes thicker over time. Water condensing in the form of a film might be a disadvantage since it extends the water–solid interface covering the entire surface of the collector and resulting in three major consequences that can be foreseen: (1) a larger interfacial force that prevents water from falling and remarkably increases the critical mass; (2) a higher evaporation rate of the condensed water that increases the time needed to reach the critical mass; and (3) a larger thermal resistance from the water film blocks heat exchange between the water vapor and the collector surface and thus affects the condensation performance of the surface.

Although the regular Al foil is less hydrophilic, its water adhesion is still considerably strong, leading to two major drawbacks: (1) the critical mass of the water droplet on regular Al is larger than that on the superhydrophobic surface. (2) A small amount of condensed water will always be left in the path of a fallen droplet. Both of these disadvantages are unfavorable to the regeneration of the collector surface. However, in the case of the superhydrophobic surface, two adjacent cycles are highly overlapped: during the coalescence phase in the preceding cycle, the surface started regeneration and the nucleation of new droplets of the succeeding cycle can be seen in Figure 5k. This functionality is absent in the untreated Al (Figure 5c). Faster release of the water droplets from the superhydrophobic Al surface and more frequent nucleation of new droplets are attributed to a significantly smaller liquid–solid interface. In contrast, water droplets on both untreated Al and superhydrophobic Al have to coalesce with the neighboring ones until their mass exceeds the critical value.

The efficiency of water harvesting can be substantially enhanced if the discrepancy between the superhydrophilic and superhydrophobic regions is utilized. Opposite to the completely superhydrophobic and completely superhydrophilic samples, the hybrid sample builds up a concentration gradient of water vapor between the superhydrophobic and superhydrophilic regions in the gaseous phase (Figure 5q). When a small disturbance takes place, water droplets condensed in the peripheral regions can be repelled from the superhydrophobic region and get collected by the superhydrophilic region (Figure 5r). A water droplet growing through coalescence in the superhydrophobic regions of the hybrid collector gets efficiently harvested by the superhydrophilic regions before its size reaches the critical mass. Therefore, the maximum size of the water droplet (∼1 mm diameter) in the superhydrophobic region of the hybrid collector (Figure 5p) is much smaller than the critical size (∼3 mm) of the droplet on the completely superhydrophobic surface (Figure 5l). This functionality accelerates water accumulation in the superhydrophilic region, so that the superhydrophobic region achieves a fast regeneration (Figure 5p). Consequently, the surface of the hybrid collector can be frequently refreshed, which outperforms other Al surfaces.

The water harvesting rate of a single repeating unit (*R*_0_) of the superhydrophilic pattern can be expressed as
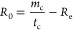
3where *m*_c_ is the critical mass of the water droplet that can be constrained within the repeating unit, *t*_c_ is the length of a regeneration cycle (the time needed for the mass of the condensed water in the droplet to reach *m*_c_), and *R*_e_ is the evaporation rate of water within the unit. Based on eq 3, to optimize the performance of a hybrid collector, the repeating unit needs to have a higher *m*_c_ to *t*_c_ ratio and a lower *R*_e_. Additionally, since *t*_c_ and *R*_e_ are both functions of temperature and relative humidity, *R*_0_ indirectly depends on temperature and relative humidity. From an energy conservation point of view, the collector consumes the least amount of energy for cooling if it is designed to work just below the dew point. Therefore, the shape of the repeating unit was optimized for dew point operation at which condensation reaches dynamic equilibrium with evaporation, and thus *R*_e_ in eq 3 can be neglected.

**Figure 6:**
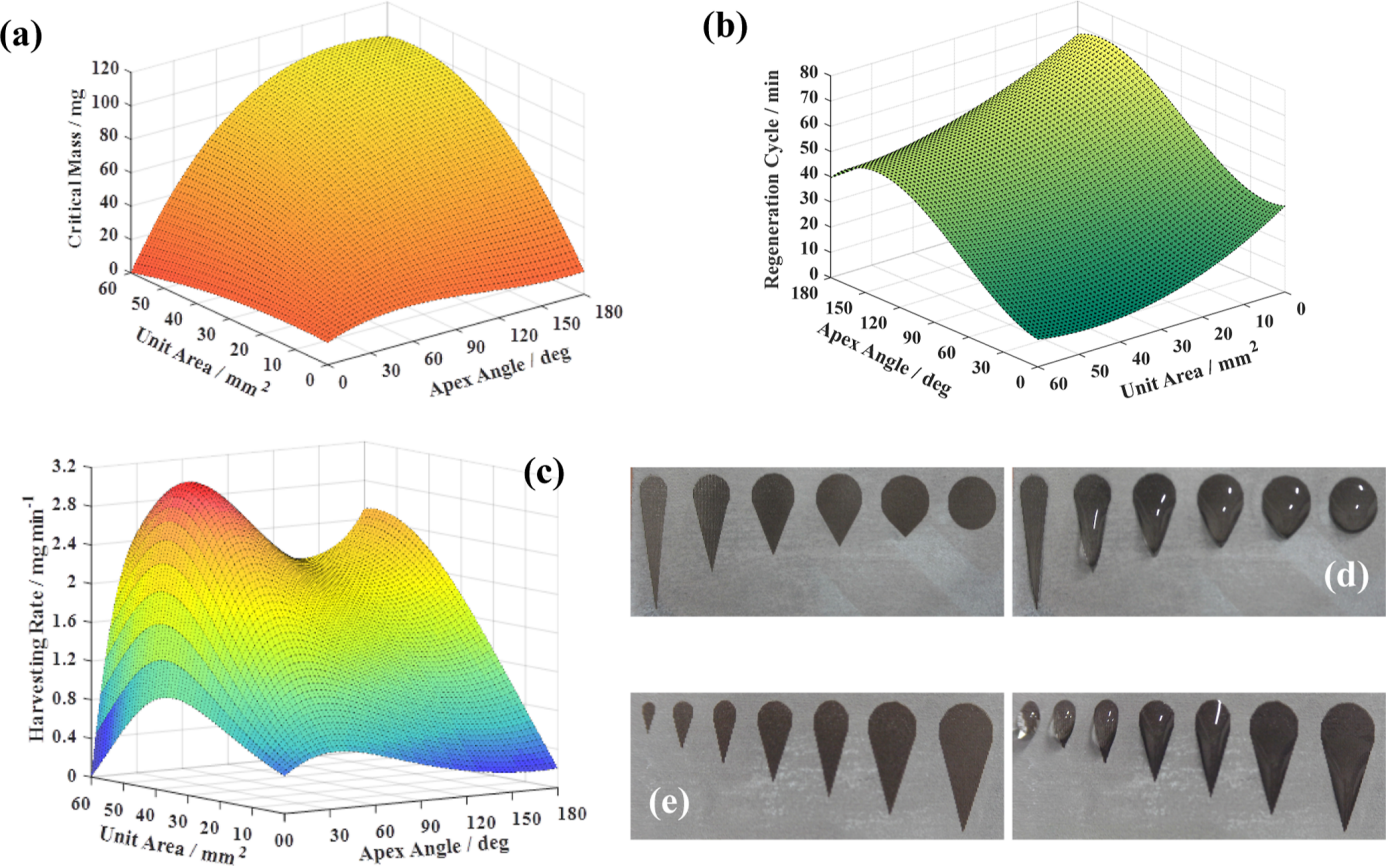
Dependency of (a) critical mass of water that can be constrained within one repeating unit of the pattern, (b) length of a regeneration cycle, and (c) water harvesting rate of one superhydrophilic unit on the area and the apex angle of the repeating unit. Photo showing before (left) and after (right) putting the same amount of water onto the teardrop-shaped superhydrophilic units with (d) apex angles of 15, 30, 45, 60, 90, and 180° and a constant area of 45 mm^2^, and (e) area of 3.14, 7.07, 12.57, 19.63, 28.27, 38.48, and 50.27 mm^2^ and a constant apex angle of 35°.

To determine *R*_0_, *m*_c_, and *t*_c_ of the superhydrophilic units, areas and apex angles of different superhydrophilic patterns were measured. Thanks to galvanometer laser processing, teardrop-shaped superhydrophilic units with areas of 3.14, 7.07, 12.57, 19.63, 28.27, 38.48, and 50.27 mm^2^ (corresponding to the circular part of the teardrop shapes with diameters ranging from 1 to 4 mm with an interval of 0.5 mm) and apex angles of 15, 30, 45, 60, 90, and 180° could be easily produced. The resulting *m*_c_ and *t*_c_ values were fitted with polynomials to interpolate as well as extrapolate, and the squares of multiple-correlation coefficients of the fitted model were 0.985 and 0.983. *R*_0_ was calculated by dividing *m*_c_ by *t*_c_. The mapped three-dimensional graphs of *m*_c_, *t*_c_, and *R*_0_ with the area and apex angle of the unit as independent variables are shown in Figure 6.

It can be seen from Figure 6a that the value of *m*_c_ grows with the area and apex angle of the superhydrophilic unit. The water affinity of the superhydrophilic unit favors the water condensation but also constrains the condensed water within the unit. It has been proven that a superhydrophilic pattern with an apex angle can create a surface tension gradient that drives the droplet toward the opposite side of the apex.^46^ By reducing the apex angle of the unit, the weight distribution of the droplet becomes less even and the deformation of the droplet becomes more pronounced (Figure 6d), which explains that smaller apex angles are more capable of tearing the droplet off from the surface. However, it might be counterintuitive that a smaller superhydrophilic unit is accountable for a longer *t*_c_, as suggested by Figure 6b. This is because units with different areas correspond to different *m*_c_, a small area helps the droplet to maintain a semi-spherical shape, while the gravitational force has a stronger impact on the shape of the droplet constrained within a larger unit. The larger gravitational impact on the droplet shape brings a larger displacement of the center of gravity (Figure 6e). Additionally, because of the severer deformation, the upper part of the droplet contributes less to the interfacial force. Furthermore, since the condensation rate is positively correlated to the area of the interface between liquid and gas phases, i.e., the surface area of the droplet, a more pronounced deformation of the droplet will result in a larger surface area of the droplet, leading to a higher condensation rate. All of the above reasons account for the shorter *t*_c_ of the units with larger areas.

**Figure 7:**
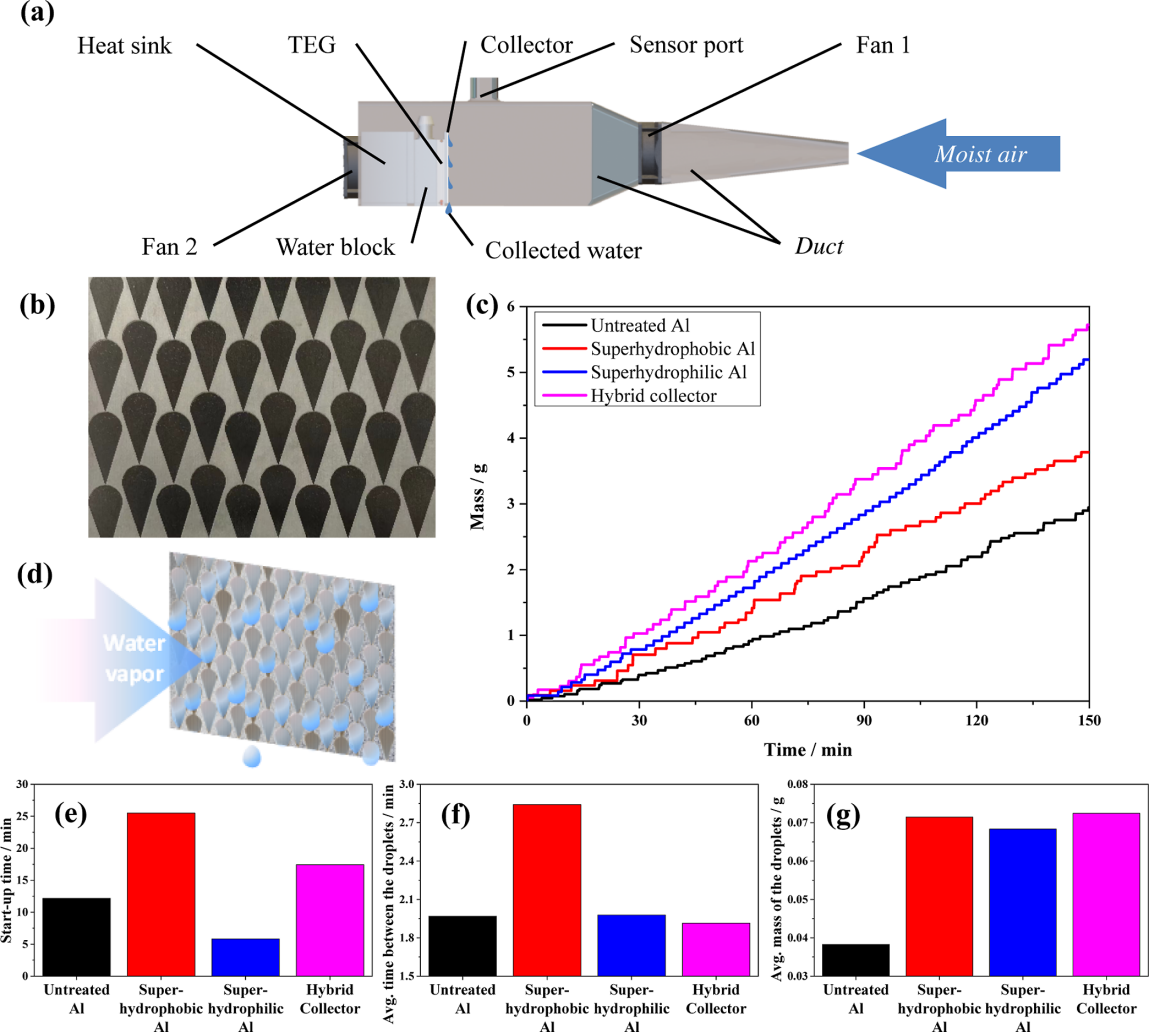
(a) Schematic setup for measuring water harvesting performances of different collectors. (b) Photo of a hybrid collector. (c) Water harvesting rates of untreated Al, complete superhydrophilic Al, complete superhydrophobic Al, and the hybrid collector. (d) Schematic illustration of the water harvesting process of the hybrid collector. (e) Start-up time, (f) average time interval between two successive droplets, and (g) average mass of the collected droplets of different collectors.

Searching through possible combinations of the area and apex angle, only a single maximum of *R*_0_ of 3.08 mg min^–1^ has been found (Figure 6c). By calculating the extreme of the curved surface, we obtained the optimum area and apex angle, which are 45.11 mm^2^ and 35.06°, respectively. The approximated values, 45 mm^2^ and 35°, were used as the dimensional parameters in fabricating the superhydrophilic units of the designed hybrid collector. The optimized superhydrophilic units were fs-laser written directly on the superhydrophobic background, and the water harvesting performance of the hybrid collector was measured using the setup shown in Figure 7a. The array of superhydrophilic patterns was designed to be vertically parallel strings of repeating units (Figure S2c). The spatial period of the strings and the spatial period of the units within one string are 5.97 and 12.91 mm (the width and height of a unit), respectively. Every other string was staggered 6.47 mm (half the height of a unit) to fully make use of the surface while reducing the interference between neighboring units. A photo of the fabricated hybrid collector is shown in Figure 7b.

### 2.4 AWH Performance of Different Collectors

Water harvesting rates of untreated Al, complete superhydrophilic Al, complete superhydrophilic Al, and the hybrid collector were measured to provide a convenient comparison of the harvesting characteristics of different samples. The results can also be seen as the performance of the collectors in a foggy environment. After being cooled down to 7.5 °C (close to the dew point), all collectors experienced a short period of starting-up, which is the time needed for the water vapor to condense and the condensed droplets to coalesce before reaching critical mass. It can be found that the order of the starting-up times follows the order of average hydrophobicity (Figure 7e). The superhydrophilic collector has the shortest starting up time of 6 min because its entire surface area is suitable for water condensation. The regular untreated Al has the second shortest (12 min) starting up time. Since over 41% of the surface area is superhydrophobic, the hybrid collector comes third with a starting up time of 17 min. The superhydrophobic Al exhibits the slowest starting up which takes 26 min. As presented in Figure 7c, the masses of water collected by all collectors show linear growth which is indicative of stable harvesting rates after starting up. As predicted, the hybrid collector shows the best performance of 0.85 kg m^–2^ h^–1^. The harvesting rates of complete superhydrophilic, untreated, and complete superhydrophobic collectors are 0.77, 0.56, and 0.44 kg m^–2^ h^–1^, respectively. The time interval (2.8 min) between two water droplets falling into the reservoir is significantly longer for the complete superhydrophobic collector (Figure 7f), which is clearly due to the water-repelling nature of the surface that makes unfavorable conditions for the nucleation and growth of the water droplets. The superhydrophilic collector possesses characteristic filmwise condensation behavior of seemingly identical droplet sizes, which is due to the fact that the mass of the droplet on the complete superhydrophilic collector is affected by the area of the entire collector as described in Section 2.3. The hybrid collector carries the advantage of fast condensing and frequent refreshing inherited from the superhydrophilic collector as well as the advantage of easier removal of water inherited from the superhydrophobic collector and therefore presents a self-pumped mechanism (Figure 7d) and exhibits the shortest average time interval between two successive water droplets (115 s) as well as the highest average mass of the droplets (72 mg). Therefore, the hybrid collector achieves a 93% AWH harvesting rate enhancement over the regular untreated Al collector.

## 3 Conclusions

In summary, we introduce a numerical design and fabricate a novel bio-inspired hybrid Al water collector for efficient AWH. The collector consists of fs-laser fabricated periodic superhydrophilic patterns on a chemically modified superhydrophobic background. Fs-laser structuring not only works as a tool to fast superhydrophilize the surface, enlarge the surface area, and direct the water flow but also introduces a self-organized microhole array at the bottom of the open microchannels that allows enhanced capillary condensation. We adopted the hybrid structure and established a significant contrast of wettability between the superhydrophobic background and the superhydrophilic pattern of the collector, a contact angle difference of ∼163°, which enables ultra-efficient droplet nucleation, coalescence, and removal phase. Concluding from the results of the experiment and simulation, we demonstrated that the inverted teardrop might be the best shape for the repeating unit of the superhydrophilic pattern, and the area and apex angle of the unit were also optimized to be 45 mm^2^ and 35° for maximum water harvesting rate. Based on the results, we have successfully optimized the hybrid collector for AWH and demonstrated a water harvesting rate of 0.85 kg m^–2^ h^–1^. Considering the stability, accessibility, flexibility, and thermal conductivity of Al, our superhydrophilic/superhydrophobic hybrid Al collector shows a great potential in solving the global water crisis by AWH.

## 4 Experimental Section

### 4.1 Sample Preparation

Untreated Al foil (AL000612, Goodfellow) with a thickness of 200 μm (similar to the thickness of common soda can walls) was cut into rectangular samples, which was performed with chemical and fs-laser treatments, as shown in Figure 1b. The foils were first chemically etched for 30 min under ultrasound in a mixed solution of concentrated hydrochloric acid (37.2%, Thermo Fisher Scientific Inc., USA) and 5% cuprous chloride (98% min, Alfa Aesar Inc., USA) with a mixing ratio of 0.25 v/v % to obtain a surface with microroughness. The ratio of the HCl/CuCl_2_ mixture solution volume to the surface area of the sample was 1.25 mL cm^–2^. After etching, the samples were cleaned with ethanol under ultrasound. To yield a superhydrophobic surface, the etched samples were treated in 0.01 mol L^–1^ stearic acid (98%, Alfa Aesar Inc., USA) ethanol solution for 24 h, followed by rinsing with ethanol and drying at ambient temperature. Finally, the as-designed superhydrophilic patterns were fs-laser-ablated on the superhydrophobic surface by programmed scanning with a SCANcube III 10 galvanometer scanner (SCANLAB GmbH, Germany) with a scanning speed of 5 mm s^–1^. The laser was generated by an Astrella-USP-1K ultrafast Ti/sapphire amplifier system (Coherent Inc., USA) with the wavelength centered at 800 nm, pulse width of 35 fs, repetition rate of 1 kHz, beam quality *M*^2^ < 1.25, and an average power of 1.25 W.

### 4.2 Surface Property Characterization

The surface elevation maps and cross-sectional profiles of the samples were acquired by CLSM with a VK-9710K color 3D laser microscope (Keyence Co. Ltd., Japan). SEM was employed to investigate the surface morphology of different samples using a JSM-6480 LV scanning electron microscope (Japan Electron Optics Laboratory Co. Ltd., Japan). The water affinity of the sample surfaces was characterized by measuring the contact angles (CA) using an SL200KB (Kino Industry Co. Ltd., USA) contact angle meter with its supplementary drop shape analysis software CAST 13.5. Fourier transform infrared spectroscopy (FT-IR) was performed to analyze the chemical composition of sample surfaces on a Nicolet 6700 FT-IR spectrometer (Thermo Fisher Scientific Inc., USA). To study the wicking dynamics of the microchannels, the sample was vertically mounted and its lower end was brought in contact with the water surface in a reservoir. To investigate the droplet behavior in different phases on samples, the samples were also vertically mounted, and moist air generated by a GXZ-J617 humidifier (Shenzhen Pioneer Technology Co., Ltd., China) was sent to the surface. A 1/1.8″ format and 6.4-megapixel back-illuminated CMOS camera ASI178MM (Suzhou ZWO Co., Ltd., China) and its complementary zoom objective with a maximum magnification power of 4.5× was applied to record the microscopic videos of droplet behaviors. The setup is shown in Figure S7.

### 4.3 Simulations

The simulations of the shapes of water droplets in static state constrained within one repeating unit of the superhydrophilic pattern on a superhydrophobic background with and without considering gravity were done using simulation programs HyDro100^35^ (AIST, Japan) and Surface Evolver^47^ (University of Minnesota, USA), respectively. These programs were designed to find the surface with minimum energy by evolving the initial surface with input parameters using the hybrid energy minimization method and the gradient descent method, respectively. All simulations were done with the same set of parameters: the density and surface tension of water, contact angles of the superhydrophilic pattern, and superhydrophobic background were set to be 1 × 10^–3^ kg m^–3^, 7.2 × 10^–3^ kg s^–2^, 0, and 160°, respectively.

### 4.4 Water Harvesting Measurements

The water harvesting performance of the collectors was judged by measuring the mass of the water that was condensed by the collector and fell onto the balance within a certain amount of time. The experiment setup is shown in Figure 7a. We designed and 3D printed a duct to perform the water harvesting experiments. Two fans were placed both in front of (Fan 1) and behind (Fan 2) the sample so that we could adjust the velocity of the airflow inside the duct. A thermoelectric cooler (TEC) was adhered to the backside of the sample, and the backside of the TEC was attached to a water-cooled heat sink. Thermal conductive paste (OMEGATHERM, Omega Engineering Inc., USA) was applied to fill in the small gaps within the TEC module (between TEC, heat sink, and Fan 2) to improve heat transfer. The room and the vapor temperatures were kept at 20 °C. Surface dry-bulb temperature as well as the relative humidity around the collector was monitored by an SHTC1 multifunctional sensor (Sensirion AG, Switzerland) throughout the experiment. Both the duct and the TEC module were treated using the as-mentioned stearic acid solution to reduce vapor condensation on their surfaces. Samples were cut into small squares with the size of 6 × 4.5 cm^2^ (same size as the cool side of the TEC module) and placed inside the duct perpendicular to the airflow. Moist air was generated using the above-mentioned humidifier with a volume flow rate of 35 mL h^–1^. The humidifier was placed beneath the duct, and the moist air was sent into the duct through a hole at the bottom of the duct behind Fan 1. Velocity of the incident moist air was accelerated to 0.1 m s^–1^ by the fans.

An indicator, water harvesting rate (*R*), is used in this paper. Water harvesting rate is the mass of water harvested in a given time per surface area of the collector and was measured in an environment with flowing moist air to mimic the actual working condition. This indicator was commonly used to characterize the harvesting performance of difference collectors and its expression is shown in eq S1. Clausius–Clapeyron relation (eq S2) is used to derive the expression of dew point (*T*_D_, in Kelvin) of the given ambient temperature (*T*, in Kelvin) and relative humidity (RH, in percentage), which is shown as follows^48^
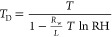
4where the specific gas constant for water vapor (*R*_w_) and latent heat of evaporation for water (*L*) are 461.52 J kg^–1^ K^–1^ and 2.5 × 10^6^ J kg^–1^, respectively. Since *T* and RH were kept at 20 °C and 44%, respectively, the sample was cooled right below the dew point, which was calculated to be 7.5 °C using the above equation. Water harvesting rates were measured by cooling the collector to dew point and with the humidifier turned on. The condensed water on the sample surface eventually fell onto a reservoir on a SMB-60 semi-micro balance (Nevada Weighing LLC, USA), which was connected to the computer to continuously record the mass increment.

## Supporting Information Available

Comparison of the related designs based on selective laser ablation, microscopic images of the hybrid Al collector, schematic illustrations of the critical receding and advancing angles of a droplet on a vertical surface, schematic illustrations of the teardrop shape superhydrophilic unit, schematic illustrations of the arrangement of superhydrophilic array, HyDro100 simulation results, CLSM surface elevation maps and cross-sectional profiles of the laser-ablated Al samples, schematic illustration of a stearic acid molecule attached to Al, FT-IR spectra of Al samples, photos reflecting the wicking behavior of water on Al samples, schematic setup for recording videos of droplet behavior, definition of water harvesting rate, derivation of expression of the density of humid air, and equilibrium vapor pressure and the volume of the condensed water in a conical pore (PDF)
