# Supplementary material for: Nature-Inspired Surface Engineering for Efficient Atmospheric Water Harvesting
Source: ACS Sustain Chem Eng. 2023 Jul 18;11(30):11019–31. doi: 10.1021/acssuschemeng.3c00760 (PMC10394688; doi:10.1021/acssuschemeng.3c00760)
Supplement: Supplementary file 1 — sc3c00760_si_001.pdf [file sc3c00760_si_001.pdf]

Supporting Information

Nature-Inspired Surafce Engineering for Efficient Atmospheric Water Harvesting

Zihao Li[a, b], Luheng Tang[a], Hanbin Wang[a], Subhash C. Singh[a, *], Xiaoming Wei[b], Zhongmin Yang[b, *], Chunlei Guo[a, *]

[a] *The Institute of Optics, University of Rochester, Rochester, NY 14627, United States*

[b] *School of Physics and Optoelectronics, South China University of Technology, Guangzhou 510640, China*

**Email address: ssingh49@ur.rochester.edu (S.C.Singh); yangzm@scut.edu.cn (Z. Yang); chunlei.guo@rochester.edu (C. Guo)*

Number of pages: 12

Number of figures: 7

Number of tables: 1

Table S1. Comparison of the related designs based on selective laser ablation

	Optimized for	Flow velocity of air (m s^{-1})	Volume flow rate of fog (mL h^{-1})	Surface temperature relative to dew point (°C)	Water harvesting rate (kg m^{-2} h^{-1})
**Ref. 26**	Centralization of water	< 0.1	300	-16	0.29
**Ref. 27**	Maximum surface energy and Laplace pressure gradient, Unidirectional transport	-	280	-24	1.11
**Ref. 28**	-	1.1	250 to 550	1.7 to 5.7	1.98
**This work**	Minimum interfacial force, Unidirectional transport	0.1	35	-18	0.85

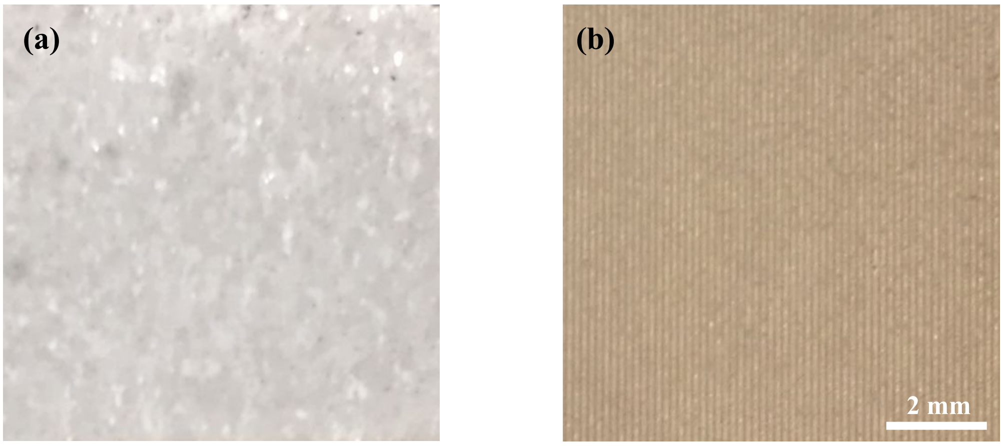

Figure S1. Zoom-in photos of the hybrid superhydrophilic/superhydrophobic Al collector. (a) The superhydrophobic background fabricated by etching and stearic acid-coating of Al foil, and (b) the superhydrophilic pattern created by fs-laser scanning of the selected superhydrophobic background.

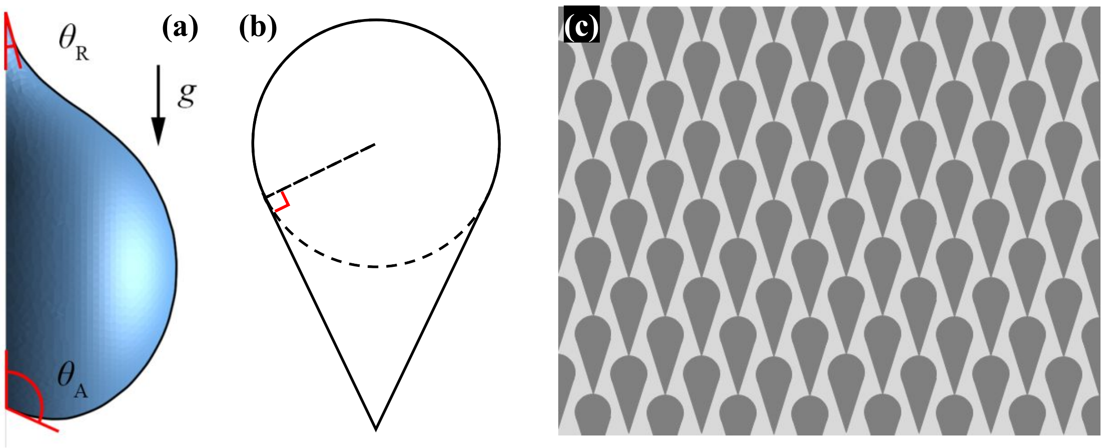

Figure S2. Schematic illustration of (a) the critical receding angle (θ_R) and the critical advancing angle (θ_A) of a droplet on a vertical surface, with the direction of gravity indicated, (b) the teardrop shape superhydrophilic unit, and (c) the arrangement of superhydrophilic array.

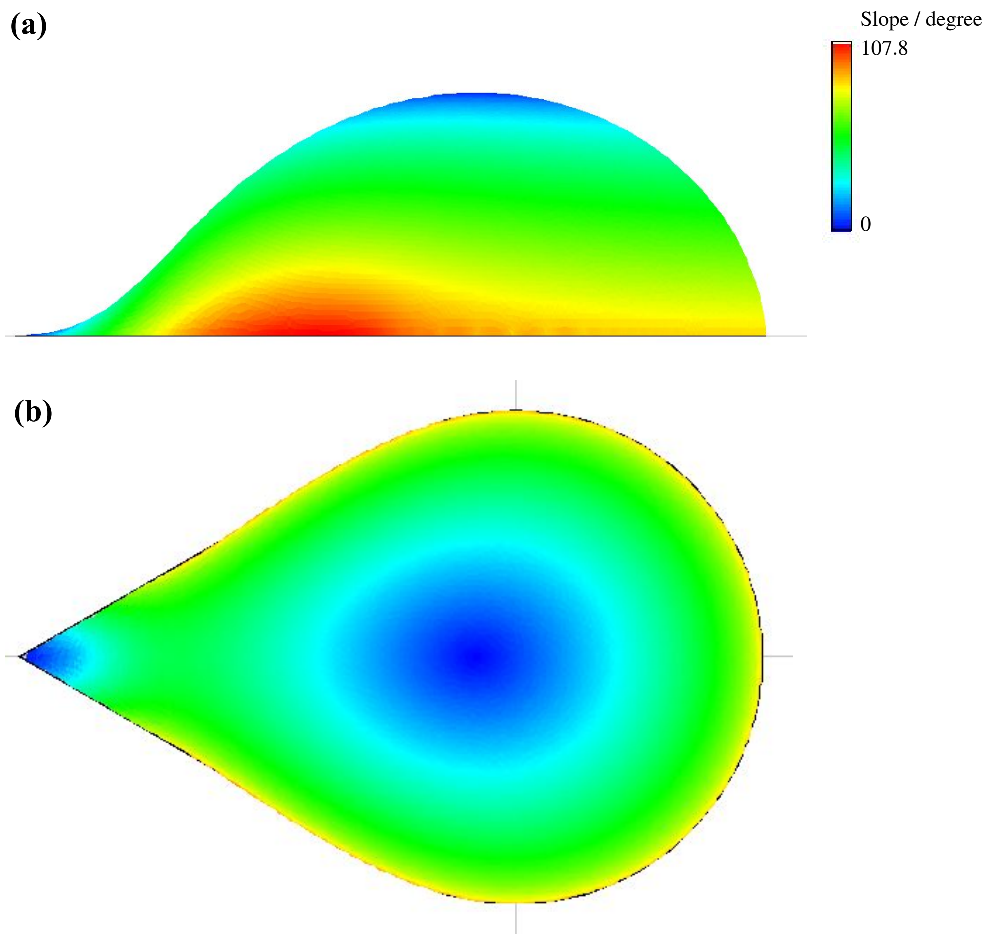

Figure S3. (a) Side view and (b) top view of the HyDro100 simulation result of a droplet being constrained within a superhydrophilic unit surrounded by superhydrophobic background. Surface of the droplet is shown in rainbow colors to represent the difference of the slope at different position. The area and apex angle of the unit are 10 mm^2 and 60° respectively and the mass of the droplet is 20 mg.

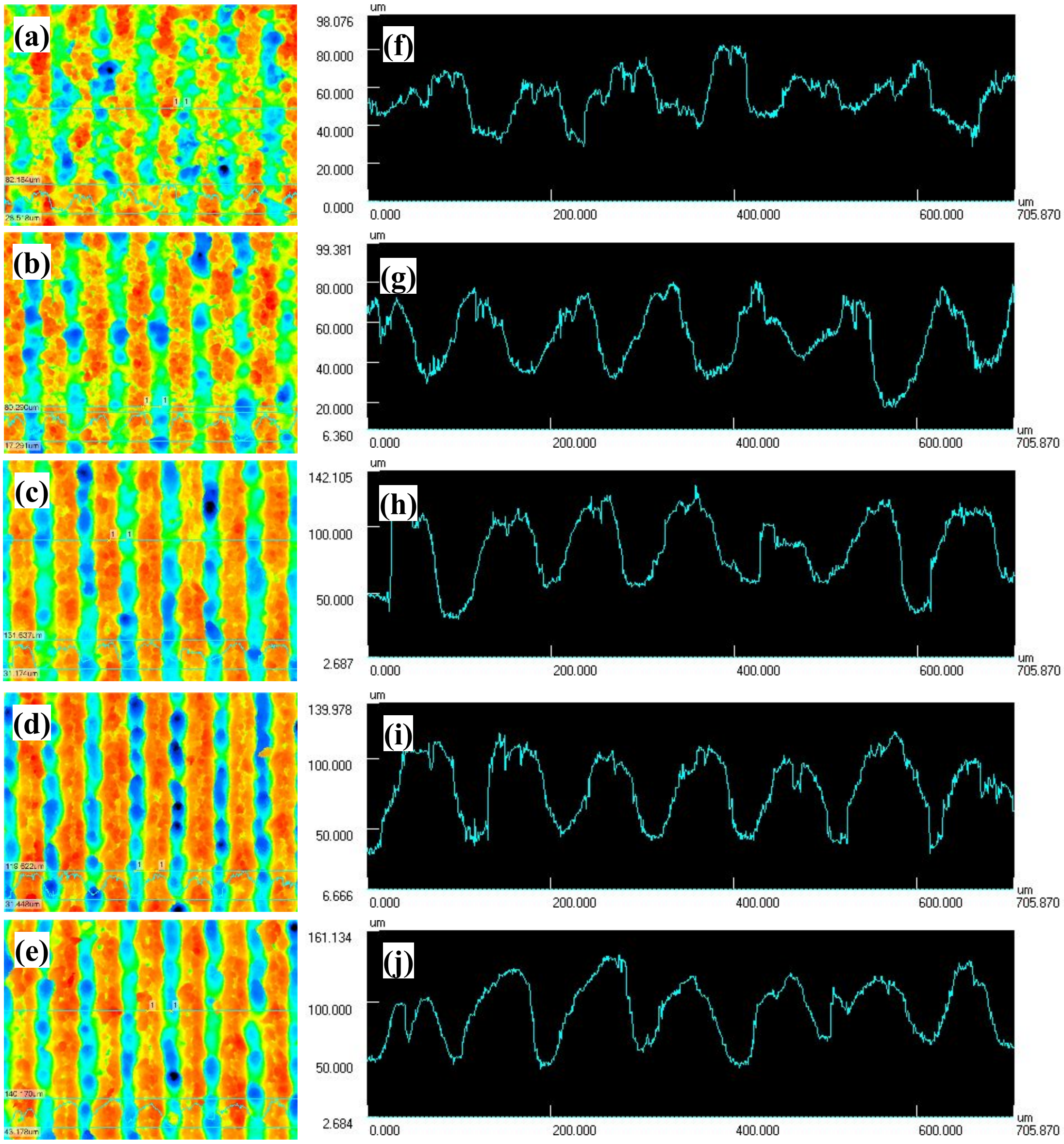

Figure S4. CLSM surface elevation maps of self-organized microhole arrays on laser-ablated Al samples with the scan number of (a) 1, (b) 2, (c) 3, (d) 4, and (e) 5. Cross-sectional profiles of fs-laser ablated Al with scan number of (f) 1, (g) 2, (h) 3, (i) 4, and (j) 5. It should be noted that in the elevation maps, different colors from blue to red only represent the relative elevation from low to high, not the absolute value.

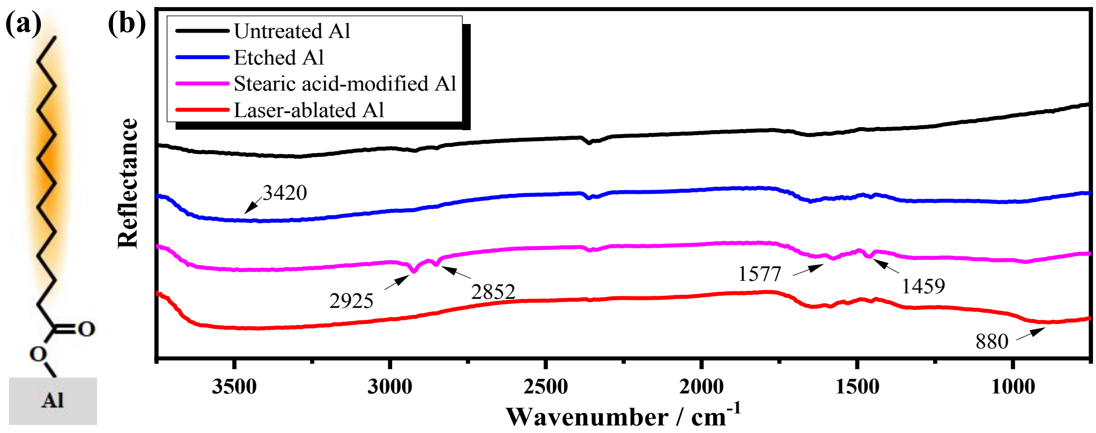

Figure S5. (a) Stearic acid molecule with a hydrophobic long alkyl chain (emphasized with orange background) attached to the Al surface through a covelant bond. (b) FT-IR spectra of different Al samples.

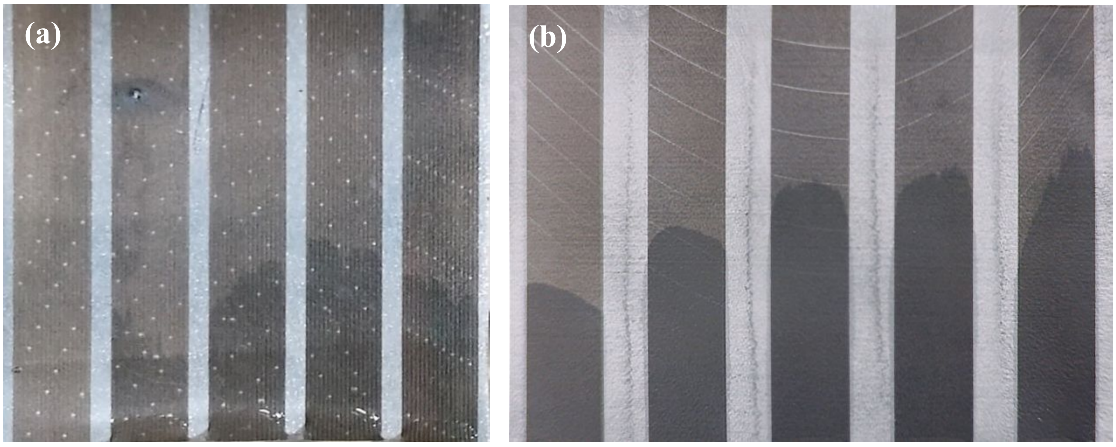

Figure S6. Photos reflecting the wicking behavior of water on Al samples with (a) various microchannel line spacings of 0.1, 0.125, 0.15, 0.175, and 0.2 mm, and (b) various groove depths generated by laser scanning for 1, 2, 3, 4, and 5 times.

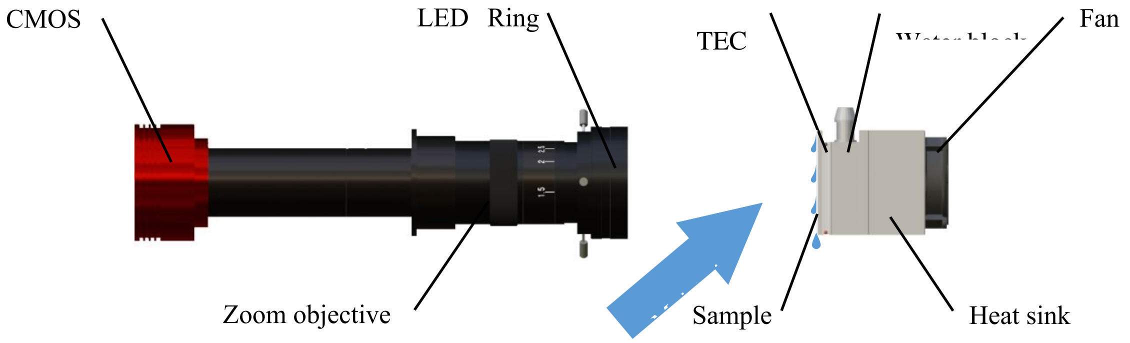

Figure S7. Schematic setup for recording videos of droplet behavior on different samples.

The expression for water harvesting rate (R)

$$R = \frac{m_{\text{receive}}}{t\text{A}} \qquad \text{Equation S1}$$

where m_{receive}, t and A are the mass of water received by the balance, the time used and the apparent surface area of the collector, respectively.

Clausius-Clapeyron equation describing the relationship between pressure (p) and temperature (T) of given substance (water) can be written as

$$\frac{\mathrm{d}p}{\mathrm{d}T} = \frac{pL}{T^2 R_{\mathrm{w}}} \qquad \text{Equation S2a}$$

where R_{w} is the specific gas constant for water vapor and L is the latent heat of evaporation for water. Assuming L is also a constant so that we can integrate Equation S3a and obtain an equation that relates two different states. In our case, the state at dew point T_{D} and a state at ambient temperature T,

$$\ln\frac{p_{\mathrm{D}}}{p} = \frac{L}{R_{\mathrm{w}}}\left(\frac{1}{T} - \frac{1}{T_{\mathrm{D}}}\right) \qquad \text{Equation S2b}$$

where p and p_{D} are the vapor pressure given temperature T and dew point T_{D}, respectively. By substituting p/p_{D} with relative humidity (RH, in percentage) according to the definition of the relative humidity (the ratio of partial water vapor pressure p_{w} and saturation water vapor pressure p_{s}), dew point can be expressed as a function of T and RH.

According to the definition of specific humidity q, it can be approximated as mixing ratio ω. Since $RH = \omega/\omega_{\mathrm{s}}$, q can be expressed using RH, we have

$$q \approx \omega_{\mathrm{s}}\, RH \qquad \text{Equation S3a}$$

Since saturation water vapor mixing ratio ω_s is the ratio of the mass of water in saturated air and mass of dry air, by combining the ideal gas law,

$$q = \frac{M_w p_s}{M_d(p_0 - p_s)} RH \approx \frac{M_w p_s}{M_d p_0} RH \qquad \text{Equation S3b}$$

where p_0 is the sea-level atmospheric pressure (one standard atmosphere of 101,325 Pa was used in this paper), and molar mass of dry air (M_d) and molar mass of water vapor (M_w) are 28.96 g mol^{-1} and 18.02 g mol^{-1}, respectively.

Integrating and rearranging Equation S2a and replace p with p_s, we will obtain the expression of p_s at any given temperature T

$$p_s = p_{s,0}\, e^{\frac{L}{R_w}\left(\frac{1}{T_0} - \frac{1}{T}\right)} \qquad \text{Equation S3c}$$

where T_0 is a reference temperature (here the triple point temperature of water, 273.16 °C is used) and $p_{s,0}$ is the saturation vapor pressure at T_0 (611.2 Pa). Therefore, T_D as a function of T and RH can be simplified as Equation 4 in the main manuscript.

The density of humid air (ρ) with volume V made up of dry air and water vapor with masses of m_d and m_w respectively, can be expressed as follow using the ideal gas law,

$$\rho = \frac{m_d + m_w}{V} = \frac{p_d}{R_d T} + \frac{p_w}{R_w T} \qquad \text{Equation S4a}$$

where R_d is the specific gas constant for dry air and equals to $1000R_0/M_d$ ($\overline{R}$ is the universal gas constant with the value of 8.3145 J mol^{-1} K^{-1}). By definition of the relative humidity,

$$\rho = \frac{p_0}{R_\mathrm{d}T} - \frac{M_\mathrm{d} + M_\mathrm{w}}{10^3\,\bar{R}T} p_\mathrm{s}RH \qquad \text{Equation S4b}$$

By combining Equation S3c, we can get the expression of ρ at given T and RH.

The Kelvin equation governs equilibrium systems involving meniscus and is used to describe the phenomenon of capillary condensation.

$$p_\mathrm{v} = p_\mathrm{s}\mathrm{e}^{-\frac{2\bar{C}\gamma V_\mathrm{m}}{\bar{R}T}} \qquad \text{Equation S5}$$

where p_v is the equilibrium vapor pressure, $\bar{C}$ is the mean curvature of the meniscus, γ is the interfacial surface tension, V_m is the molar volume of the liquid.

Consider a conical pore with a half apex angle of θ. If a meniscus can form with zero angle of contact with the wall of the pore, when the radius of curvature r reaches its maximum, the corresponding maximum volume of condensed water V_r in the pore will be[1]

$$V = \frac{\pi r}{3}\frac{(1-\sin\theta)^2}{\sin\theta} \qquad \text{Equation S6}$$

References

(1) Barrer, R.; McKenzie, N.; Reay, J. Capillary condensation in single pores. *J. Colloid Sci.* **1956**, *11* (4-5), 479-495.
